# Evaluation of occupational exposure to airborne quartz in the cutting and grinding of ceramic tiles

**DOI:** 10.1093/annweh/wxaf044

**Published:** 2025-09-16

**Authors:** Francesca Borghi, Francesca Graziosi, Silvia Contessi, David C Christiani, Francesco Decataldo, Deborah Glass, Francesco S Violante

**Affiliations:** Occupational Medicine Unit, Department of Medical and Surgical Sciences, Alma Mater Studiorum University of Bologna, Bologna, Italy; Occupational Medicine Unit, Department of Medical and Surgical Sciences, Alma Mater Studiorum University of Bologna, Bologna, Italy; Occupational Medicine Unit, Department of Medical and Surgical Sciences, Alma Mater Studiorum University of Bologna, Bologna, Italy; Harvard Medical School and Harvard T.H. Chan School of Public Health, Boston, MA, United States; Occupational Medicine Unit, Department of Medical and Surgical Sciences, Alma Mater Studiorum University of Bologna, Bologna, Italy; Monash School of Public Health and Preventive Medicine, Melbourne, VIC, Australia; Occupational Medicine Unit, Department of Medical and Surgical Sciences, Alma Mater Studiorum University of Bologna, Bologna, Italy; Division of Occupational Medicine, IRCCS Azienda Ospedaliero-Universitaria di Bologna, Bologna, Italy

**Keywords:** benchtop, occupational health, personal exposure, PPE, respirable crystalline silica

## Abstract

Crystalline silica was categorized by the International Agency for Research on Cancer as a known human carcinogen. Activities related to the processing of ceramic tiles, releasing crystalline silica, may vary considerably in terms of hours worked per day and days worked per week. This variability could be particularly high for craftsmen who process ceramic materials directly on-site during installation. The aim of this study is to measure the likely exposure to respirable crystalline silica (RCS) during ceramic tiles installation, evaluating the exposure to RCS of workers processing these tiles. Exposure assessments to RCS were conducted via both fixed-site and personal sampling for 2 working hours. The measured concentrations were calculated as 8-h time-weighted average (TWA) exposures, assuming no further RCS exposure in the time period. The permitted exposure time, not to exceed the occupational exposure limit (OEL) value, was then calculated also considering the assigned protection factor of selected respiratory protective equipment. The results of this study, considered as a worst-case simulation, show that, during the processing of ceramic tiles releasing RCS, the worker exposure can be very high (up to 240.9 µg/m^3^), exceedance of several OELs, including the European OEL of 100 µg/m³.

Even working for a few hours a day, the RCS 8-h TWA OEL is likely to be exceeded. Inhaled exposure concentrations can be reduced by using appropriate respiratory protection, by a factor equal to 10 or 20. The assumption of this work was that (i) the cutting/grinding times are not always necessarily equal to 2 h and that (ii) these processes are not characterized by pre-established and continuous processing times. For these reasons, it is important to carefully evaluate the duration of exposure to RCS during the various tasks/activities performed, as these may vary depending on different factors.

**What’s Important About This Paper?**

This study characterized workers’ exposures to respirable crystalline silica (RCS) associated with cutting and grinding of ceramic tiles, which contained 7.9%–30.5% quartz. Exposures were highly variable but quickly exceeded occupational exposure limits, requiring the use of control technologies. High RCS exposures among countertop workers are not limited to engineered stone but can also be encountered with ceramic tiles.

## 1. Introduction

Crystalline silica, in the form of α-quartz, is a common mineral in the earth’s crust (Rey-Brandariz et al. 2023). Exposure to respirable crystalline silica (RCS) can occur especially in occupational settings (McLean et al. 2017), including (i) mining; (ii) processing of mineral materials; (iii) foundries; (iv) construction; and (v) glass, ceramics, bricks, and tiles industry (Scarselli et al. 2014; Rey-Brandariz et al. 2023). Exposure to RCS occurs mainly where materials that contain crystalline silica are mechanically processed through, for example, cutting, grinding, crushing, drilling, or abrasive blasting (McLean et al. 2017; Tuomi and Lyyränen 2024).

Inhalation of RCS can present a significant health hazard (NIOSH 2002) and the adverse health effects of occupational exposure to RCS are well known. Numerous studies have shown that RCS can lead to silicosis, which is a progressive and irreversible nodular fibrotic lung disease. It can develop as an (i) acute form after massive exposure to RCS, causing severe lung disruption that can lead to respiratory failure; (ii) an accelerated form, similar to chronic silicosis that develops rapidly in subjects with high exposure, with onset of disease within 5 to 10 years after initial exposure; (iii) chronic form, which occurs 10 to 30 years after continued exposure to lower levels of quartz (McLean et al. 2017; Hoy et al. 2023; SafeWork Australia 2022a; Tuomi and Lyyränen 2024). Silicosis can be also considered a generalized systemic disease that originates in the lung and often affects other systems and organs (Ramkissoon et al. 2022). Recently, several studies demonstrated associations between exposure to RCS and other autoimmune-related effects, such as rheumatoid arthritis, scleroderma and systemic lupus erythematosus, and chronic kidney disease (McLean et al. 2017), as well as an increased risk of developing other respiratory diseases (e.g. chronic bronchitis and chronic obstructive pulmonary disease) and other diseases including tuberculosis and lung cancer (Hoy et al. 2023). Crystalline silica, inhaled in the form of quartz or cristobalite, was categorized by the IARC (International Agency for Research on Cancer) as a known human carcinogen—Group 1, in 1997 (IARC 1997). A recent evaluation identified that (i) 4 additional deaths of lung cancer could be expected per 100 000 workers (4 × 10^−5^), for 40 yr of occupational exposure, equal to 0.00038 mg/m^3^ and (ii) 4 additional deaths of lung cancer could be expected per 1 000 workers (4 × 10^−3^), for 40 yr of occupational exposure, equal to 0.0363 mg/m^3^ (Boers 2024).

Most countries have adopted RCS occupational exposure limits (OELs), typically expressed as an 8-h time-weighted average (TWA). As reported by Hoy and collaborators (Hoy et al. 2023), RCS international exposure limits are generally in the range of 0.05 to 0.1 mg/m^3^. In Europe, Directive 2004/37/EC sets an OEL TWA limit equal to 0.1 mg/m³ (European Commission 2004). However, this limit does not protect workers adequately against silicosis or the carcinogenic effects of RCS (Salamon et al. 2021). In the United States, the Occupational Safety and Health Administration (OSHA) has set a permissible exposure limit (PEL) for RCS of 0.05 mg/m³ over an 8-h period, while an OEL equal to 0.05 mg/m³ was also set in Australia (SafeWork Australia 2022b). In Canada, the exposure limits vary between provinces, with some adopting the threshold limit value (TLV) of 0.025 mg/m³ recommended by the American Conference of Governmental Industrial Hygienists (ACGIH). The variety of exposure limits reflects the different policies and approaches to occupational health regulation globally, and how recently the exposure limit was set (Boers 2024).

However, the common goal remains the protection of workers from the risks associated with RCS, through the implementation of control measures (e.g. adequate ventilation, the use of personal protective equipment [PPE], and adequate training of workers) and the promotion of safe working practices.

Recently, an epidemic of silicosis associated with working with high RCS content engineered stone slabs lead a ban of these materials in Australia (Nogrady 2023). Artificial stone can contain 90% or more of RCS, mixed with resins and pigments (Leso et al. 2019). If this material is processed (e.g. cutting or grinding), without the use of exposure control measures, workers will be subjected to high exposure to RCS. Ceramic tiles may be substituted for engineered stone slabs as they have the same esthetic characteristics. The content of RCS of ceramic tiles is lower than that of engineered stone slabs, and thus, they are likely to emit less RCS when processed. Specifically, ceramic tiles undergo a sintering process at ~1250 °C, to bind the constituents which distinguish them from other materials made from resins and recycled fired tile scraps.

Activities related to the processing of ceramic tiles may vary considerably in terms of hours worked per day and days worked per week. This variability is high for craftsmen who carry out cutting, grinding, or other processing of ceramic materials directly on site, during installation of tiles (where adequate collective protection systems may be lacking) and not in a completely suitable environment (typically, a house or other type of building), in terms of health and safety of the worker. In these situations, it may be impossible, or at least very difficult, to control RCS exposure by means of dust extraction or abatement systems such as wet work conditions. Furthermore, actual working time for these operations is unpredictable. These uncertainties make it difficult to estimate the exposure to RCS of artisans/craftsmen, a critical factor for assessing occupational risks and implementing appropriate safety measures.

In this study, we measured the likely exposure to RCS during ceramic tile installation by measuring the exposure to RCS of workers processing these tiles.

## 2. Materials and methods

### 2.1 Sampling strategy

We sampled personal exposure to RCS during the simulation, in terms of activities duration and location, of kitchen/bathroom countertops processing outdoors, including cutting and grinding tasks. Although tile processing may be performed indoors in specialized facilities, cutting and grinding tasks are also frequently carried out on-site, including outdoors, especially during installation phases where *ad hoc* adjustments are needed. The processed tiles, which look like stone slabs (dimensions: 80 × 160 cm; thickness: 12 and 20 mm), were Black—B (traditional spray dried powder, a mix of feldspars and clays [maximum percentage by weight of the minerals presents in bulk samples: 30.5]) and White—W (white spray dried powder, a mix of glass, feldspar, and clays [maximum percentage by weight of the minerals presents in bulk samples: 27.2]). Details on the composition of the materials are reported in Results section—Table 3. Different manual equipment without ventilation system was used for cutting and grinding in this study: diamond cup—Ø 27 mm (drilling); diamond cutting disc—Ø 115 mm (linear cut); superflex abrasive disc—Ø 115 mm; grain—60 (edge finish and coarse chamfering); diamond flap blade—Ø 115 mm; and grain—120 (edge finishing and fine chamfering).

Exposure assessments to RCS were conducted via (i) both fixed-site/environmental sampling positioned at 1.5 m from the workbench and at a height of about 1.60 m above the ground, and (ii) personal subject’s breathing zone sampling, for 2 working hours (Table 1).

**Table 1.** Sampling strategy—sampling time: 2 h. B: Black, and W: White. Each individual sample was collected while processing a single tile type and thickness.

Material	Personal samples (total *N*)	Environmental samples (total *N*)
*B12 + 20 mm* ^*a*^	*8 (cutting: 4; grinding: 4)*	*16*
B12 mm	4 (cutting: 2; grinding: 2)	8
B20 mm	4 (cutting: 2; grinding: 2)	8
*W12 + 20 mm* ^*a*^	8 (cutting: 4; grinding: 4)	*16*
W12 mm	4 (cutting: 2; grinding: 2)	8
W20 mm	4 (cutting: 2; grinding: 2)	8

^a^The rows in italics indicate the total number of samples collected for each material (black and white), combining both 12- and 20-mm thicknesses. No combined processing of different thicknesses or materials was performed during a single sampling session.

**Table 2.** Summary of personal and environmental exposure measurements collected during 2-h sampling sessions. Results are grouped by tile material, thickness, and task performed (cutting or grinding). Each individual sample was collected while processing a single tile type and thickness.

RCS [µg/m^3^]								
			*N*	Min.	Mean	Median	Max.	SD; GSD
Personal samples	Material	*Total by material* ^*a*^						
		*W12 mm + W20 mm* ^*a*^	*8*	*8.2*	*97.5*	*98.9*	*223.1*	*62.0; 2.7*
		*B12 mm + B20 mm* ^*a*^	*8*	*16.0*	*384.4*	*312.3*	*889.3*	*287.5; 3.4*
		Detailed						
		W12 mm	4	99.8	140.5	119.6	223.1	49.3; 1.4
		W20 mm	4	8.2	54.5	55.9	98.0	39.3; 2.8
		B12 mm	4	16.0	234.5	192.8	536.3	188.9; 3.7
		B20 mm	4	123.6	534.3	562.2	889.3	291.0; 2.1
	Activity	Cutting	8	16.0	333.1	164.5	889.3	310.4; 3.5
		Grinding	8	8.2	148.7	128.5	416.3	119.8; 3.3
	**Total** ^b^		**16**	**8.2**	**240.9**	**128.5**	**889.3**	**252.7; 3.6**
Environmental samples	Material	*Total by material* ^*a*^						
		*W12 mm + W20 mm* ^*a*^	*14*	*8.1*	*35.7*	*24.0*	*148.6*	*35.8; 2.3*
		*B12 mm + B20 mm* ^*a*^	*16*	*18.3*	*45.8*	*37.8*	*89.1*	*22.8; 1.6*
		Detailed						
		W12 mm	8	8.1	35.3	37.2	61.4	18.2; 2.1
		W20 mm	6	9.9	36.2	15.0	148.6	50.4; 2.5
		B12 mm	8	18.3	32.3	30.1	61.0	13.1; 1.5
		B20 mm	8	32.4	59.4	53.3	89.1	22.4; 1.5
	**Total**		**30**	**8.1**	**41.1**	**32.4**	**148.6**	**30.0; 2.1**

Key. *N*.: number of measurements; Min.: minimum; Max.: maximum; SD: standard deviation; GSD: geometric standard deviation.

^a^The rows in italics indicate the total number of samples collected for each material (black and white), combining both 12 mm and 20 mm thicknesses. No combined processing of different thicknesses or materials was performed during a single sampling session.

^b^Cutting: White, 20 mm (*N* = 2); White, 12 mm (*N* = 2); Black, 12 mm (*N* = 2); Black, 20 mm (*N* = 2). Grinding: White, 20 mm (*N* = 2); White, 12 mm (*N* = 2); Black, 12 mm (*N* = 2); Black, 20 mm (*N* = 2).

In total, 5 different workers participated in the study. Each personal sample represents a distinct sampling session, and in some cases, the same worker was sampled more than once, but never within the same trial. Therefore, while there were repeated measures for some individuals, the exposure conditions varied between sampling days, minimizing intra-individual bias.

Samples were taken with a cyclone sampler (Casella Higgins Dewell cyclones; TSI—for the respirable fraction), operating at a constant flow of 2.2 l/min (sampler used: AirCheck Connect—SKC [ISO 13137:2022]). Silver filters (Steriltech; 25 mm; 0.8 μm) were used as a collection substrate. The sampling flow was calibrated presampling and verified postsampling (maximum variation: ±5%) via a digital flow meter (SKC - 135EZ11). In addition to personal and fixed sampling, field blanks were sampled for each monitoring day.

Throughout the entire simulation, workers used PPE. No local exhaust ventilation (LEV) and no wet processing systems were used, to evaluate the worst-case scenario, typical of the field activities of installers.

Eight sampling of the bulk raw material was conducted on both samples and thicknesses (B12 mm; B20 mm; W12 mm; W20 mm), coring for the entire height of the processed tile.

The present project was approved by the Institutional Review Board of the University of Bologna, with protocol number 0127889.

### 2.2 Gravimetric and diffractometric analysis

Gravimetric analyses were performed pre- and postsampling, following UNICHIM 2010, 2011. Analytical determination of RCS via X-Ray Diffraction (XRD) was performed on the filter used for dust sampling, according to ISO 16258-1:2017 standard. RCS present in the dust deposited on the filters (silver filters; 25 m; 0.8 μm) was measured by applying the external standard method, which provides for the comparison of the diffractometric signal acquired on the unknown sample with calibration curves obtained with filter-samples containing known quantities of reference materials (known purity, crystallinity, and particle size—Quartz ST3911 [98.9%]). The limit of quantification (LOQ) for RCS is equal to 1.6 to 1.7 µg (6.2 to 8.6 µg/m^3^). For the analysis of the raw material, EN 13925-2:2006 method was applied. Each of the bulk samples was ground to powder and analyzed by XRD for percentage quartz content and presented as percentage of quartz by weight. Quartz analyses were performed by a laboratory accredited by ACCREDIA, the Italian Accreditation Body.

### 2.3 Statistical analysis

Average RCS exposure measurements obtained in this 2-h simulation campaign in µg/m^3^ were derived by dividing the mass of RCS in the sample by the volume of air and these values were compared with several OELs, including ACGIH TLV (0.025 mg/m³), OSHA PEL (0.05 mg/m³), and European OEL (0.1 mg/m³).

Descriptive statistics of the RCS measurement data were carried out by task performed (i.e. cutting or grinding) and by type of material.

The personal measured concentrations were calculated as 8-h TWA exposures assuming no further RCS exposure in the time period (ACGIH 2024). TWA exposure values (Table 4) were calculated using only personal sampling results, as these best represent actual worker exposure. These 8-h TWAs were compared with the ACGIH TLV, the OSHA PEL, and the European OEL. The permitted exposure time was then recalculated considering the assigned protection factor (APF) of selected respiratory protective equipment. Results regarding the period of time which an operator can perform a task so as not to exceed a certain exposure value are reported as well. The calculation of 8-h exposures was performed under the assumption of no additional exposure outside the 2-h sampling period. This approach allows for the comparison and analysis of working conditions with longer or shorter processing times relative to the sampling period, providing flexibility in assessing compliance with OELs across varying scenarios.

**Table 3.** Percentage by weight of the minerals present in bulk samples and, for % p/p of quartz, in respirable dust samples (*).

Material	Cristobalite [% p/p]	Tridymite [% p/p]	Quartz [% p/p]	LOQ
W12 mm	<0.1	<0.1	27.2 (*12.8%)	0.1
W20 mm	<0.1	<0.1	7.9 (*6.1%)	0.1
B12 mm	<0.1	<0.1	27.4 (*11.9%)	0.1
B20 mm	<0.1	<0.1	30.5 (*13.5%)	0.1

**Table 4.** Time-weighted exposure calculation, for tile cutting and grinding processes (data obtained from personal sampling).

Minutes/Day	CUTTING											
	20	30	40	60	90	120	180	240	300	360	420	480
Mean values [µg/m^3^]	13.9	20.8	27.8	41.6	62.5	83.3	124.9	166.6	208.2	249.9	291.5	333.1
Median values [µg/m^3^]	6.9	10.3	13.7	20.6	30.8	41.1	61.7	82.2	102.8	123.3	143.9	164.5
Max. values [µg/m^3^]	37.1	55.6	74.1	111.2	166.7	222.3	333.5	444.7	555.8	667.0	778.1	889.3
Minutes/Day	GRINDING											
	20	30	40	60	90	120	180	240	300	360	420	480
Mean values [µg/m^3^]	6.2	9.3	12.4	18.6	27.9	37.2	55.8	74.4	93.0	111.6	130.1	148.7
Median values [µg/m^3^]	5.4	8.0	10.7	16.1	24.1	32.1	48.2	64.2	80.3	96.3	112.4	128.5
Max. values [µg/m^3^]	17.3	26.0	34.7	52.0	78.1	104.1	156.1	208.1	260.2	312.2	364.3	416.3

Key. Green: 0–25 µg/m^3^ (<TLV ACGIH Exposure Limit); Yellow: 25–50 µg/m^3^ (<OSHA Permissible Exposure Limit); Orange: 50–100 µg/m^3^ (<European Exposure Limit); and Gray: >100 µg/m^3^.

In the following results, data were grouped by material type (White or Black) and combined across both thicknesses (12 and 20 mm). Although each individual measurement was obtained from experiment using a single thickness and material type, the grouping reflects real-world working conditions in which workers often process tiles of different thicknesses and composition/material during the same work shift.

## 3. Results

### 3.1 Descriptive statistics

**Fig. 1.**
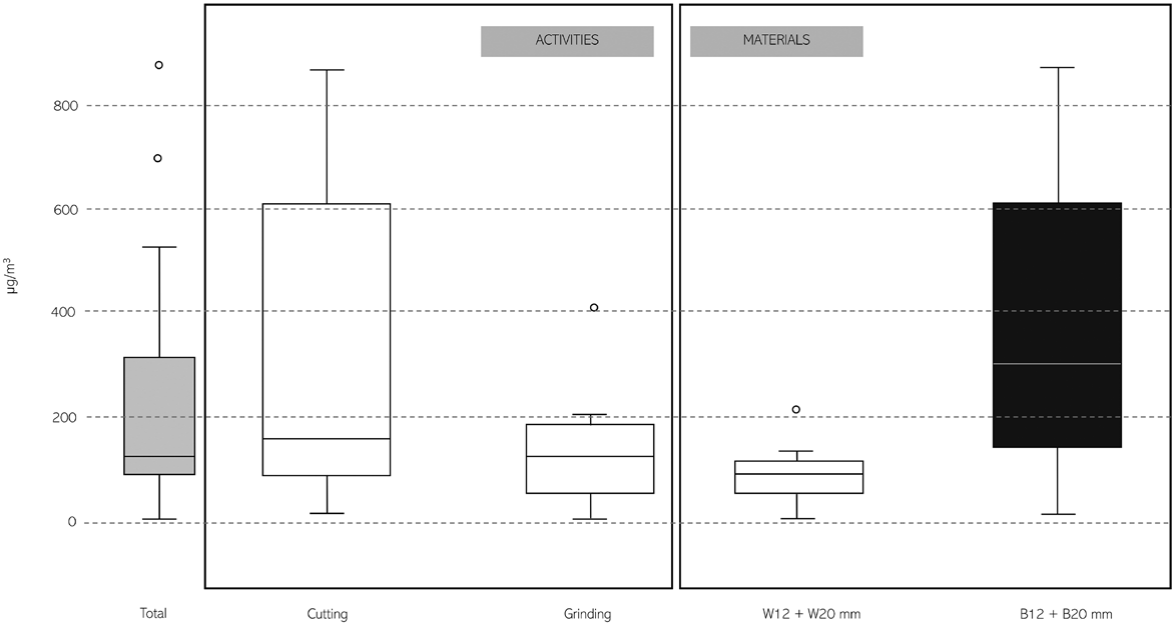
Boxplot of measured exposure concentrations of RCS (µg/m^3^). Analysis is presented divided for material and activity performed.

Table S1 reports gravimetric analyses conducted pre- and postsampling. Table 2 reports the concentrations of RCS measured during the tile processing (sampling time: 2 h; wind speed (minimum–maximum): 0.1 to 1.3 m/s), for personal and environmental samples. Figure 1 illustrates the variability in RCS concentrations associated with different tasks and materials.

Cutting tasks resulted in higher exposure levels compared with grinding, with a median RCS concentration of 164.5 µg/m³ versus 128.5 µg/m³, respectively. Similarly, the processing of black tiles produced higher airborne RCS levels than white tiles, with median concentrations of 312.3 and 98.9 µg/m³, respectively.

All blank samples (*n* = 3) were below the LOQ of 1.6 µg/m^3^.

The percentage of quartz by weight differed between bulk samples (Table 3).

### 3.2 Exposure simulation

Processing ceramic tiles in the field may require cutting and grinding for variable lengths of time, and thus, the 8-h TWA exposure may be almost impossible to predict. Table 4 shows the minutes per day that a worker can carry out tile processing before exceeding the 8-h TWA, assuming no other RSC exposure. The times vary depending on whether the mean, median, or maximum measured RCS values are used.

Using the maximum measured values, workers could cut tiles *without respiratory protection* between 13, 27, and 54 min per day without exceeding relevant OELs (ACGIH TLV, OSHA PEL, and European OELV, respectively). Tile grinding activities *without respiratory protection* could be performed for a maximum of 29, 58, and 115 min per day without exceeding relevant OELs (ACGIH TLV, OSHA PEL, and European OELV, respectively).

The correct use of masks and respirators means that the worker has potentially lower exposure to RCS. Applying an APFs equal to 10 and 20, respectively, for FFP2 and FFP3 masks (N95 and N99 in the United States) means that the worker can theoretically work for 10 or 20 times longer before exceeding the relevant OEL (Table S2).

It is possible to calculate how long an operator can perform a task, so as not to exceed a certain exposure value, considering APFs. The results of this processing are reported in Table S3. As can be seen from the table, as expected, by using appropriate PPEs, the time of exposure to RCS can increase considerably.

It is evident from Tables S3 that with the use of a FF2 (N95) respirator, a worker could perform cutting or grinding of ceramic tiles (assuming that the RCS concentration in the air equals the maximum level measured in this study) for at least 135 min (cutting) or 288 min (grinding) not being exposed to a level of RCS above 25 µg/m^3^: the times for TLVs of 50 or 100 µg/m^3^ are proportionally longer. With the use of FFP3 (N99) respirators grinding could be performed for more than 8 h and cutting could last 270 min not being exposed to a level of RCS above 25 µg/m^3^: the times for TLVs of 50 or 100 µg/m^3^ are always longer than 8 h.

## 5. Discussion

The gravimetric data presented in Table S1 show a difference between personal and environmental exposure measurements. Personal samples are characterized by a higher dust masses compared with fixed-site samples, confirming that personal monitoring better captures short-term variations and the worker’s proximity to the source. Similarly, from Table 2, it is evident that the mean results obtained from sampling performed in a fixed location (mean: 41.1 µg/m^3^) are an order of magnitude lower than the sampling conducted in the breathing zone (mean: 240.9 µg/m^3^). This expected phenomenon has been documented in several studies (Cherrie 1999; Violante et al. 2006), which consider as the main explanation the fact that personal measurements capture individual exposure dynamically, considering work behaviors and variations in the concentration of agents in time and space. Fixed locations may underestimate exposure because they do not reflect changes in worker position and are often placed at maximum exposure representative location (Kromhout et al. 1993): as reported in the literature, indeed, data derived from ﬁxed monitoring stations are unable to explain adequately the personal exposure also to airborne pollutants, especially in the short period (Cattaneo et al. 2010). It is important to underline here that, in the case of sampling performed at a fixed location, the results are not to be considered as strictly indicative of the occupational exposure, but nevertheless, they are useful for further considerations in the results’ evaluation: for compliance purposes, samples must be taken in the breathing zone.

Table 2 also shows differences in terms of exposure to RCS as a function of the material treated and its thickness. Probably, this may be due to (i) the intrinsic characteristics of the materials and to their (ii) thickness, which influences the preparation time (i.e. backing in a high-temperature kiln) of the material itself (e.g. materials with a thickness equal to 20 mm are fired for 4 h; material with a thickness of 12 mm are fired for 2 h). In addition, exposure to RCS during benchtop processing (cutting and grinding) is potentially indirectly influenced by the characteristics of the starting material: the hardness of the material, for example, may affects the type of cutting performed (harder materials require a more aggressive cut, which can generate a greater amount of dust). Also, the thickness of the material can affect the processing time and therefore the exposure time (processing on thicker sheets can result in a longer processing time, generating a greater amount of dust than cutting thinner sheets).

There could be variability introduced by the operators (Table 2). Although this was not specifically investigated in the present study, such variability may be influenced by several factors, including: (i) the operator’s skill and experience, which can affect the speed and precision of cutting; (ii) the specific cutting or grinding techniques adopted; (iii) the quality and condition of the tools used; and (iv) the application of control measures, such as wet methods and LEV (Nij et al. 2004; van Deurssen et al. 2014). As said, due to the limited number of personal samples collected, it was not possible to clearly assess the relative contribution of these factors (along with tile type, thickness, and task performed) to the variability in airborne RCS concentrations. The observed differences are likely the result of multiple interacting variables, and future studies with larger datasets will be needed to better characterize their individual and combined influence.

Variability in exposure is consistent with what has been reported in the literature regarding construction and task-based activities, where exposure determinants are known to fluctuate. In particular, as highlighted by Sauvé and collaborators (Sauvé et al. 2013), the assessment of RCS exposure in such environments remains particularly challenging due to the factors’ variability, such as tasks performed, materials handled, and worksite conditions. This further supports the need for detailed, task-based exposure assessments that can more accurately reflect the dynamic nature of real-world occupational environments.

Table 4 shows that even working for a few hours a day, the OEL is likely to be exceeded. The simulations consider a single task, performed for a certain number of minutes: however, a worker may perform both tasks during the same day.

It is important to underline that the calculated exposure durations reported in this study are based on the assumption, as stated, that no further RCS exposure occurs during the rest of the workday. In real-world scenarios, workers may be exposed to additional sources of silica, such as ambient dust, cleanup activities, or performing multiple tasks involving RCS within the same work shift. Therefore, the actual total exposure could be higher than estimated, and permissible durations should be interpreted as idealized upper limits under strictly controlled conditions.

Moreover, it should be noted that the assumption of exposure occurring only during active processing may not reflect actual field conditions, where airborne RCS concentrations can remain suspended even after cessation of processing activities. This may lead to the underestimation of daily total exposure. It is important to underline that the calculated exposure durations reported in this study are based on the assumption, as stated, that no further RCS exposure occurs during the rest of the workday. This assumption was adopted to simplify the interpretation of the task-based simulation results and to isolate the contribution of specific activities (cutting or grinding) to 8-h TWA exposure. The simulation was conducted outdoors in an open, nonconfined space with no subsequent dust-generating tasks, thereby minimizing potential residual or background exposure. Moreover, to ensure a conservative estimation, calculations were based on the maximum measured personal exposure levels. However, in real-world conditions, workers may continue working on-site and be exposed to additional sources of RCS (e.g. ambient dust, residual airborne particles, or other intermittent tasks involving crystalline silica) throughout the work shift. Therefore, the total daily exposure could be higher than estimated. As noted in previous studies, indeed, background concentrations present on construction sites can contribute substantially to overall exposure and, in some situations, may lead to overexposure even when individual task durations are limited (Cothern et al. 2023).

As highlighted from Table 4, exposure concentrations in the simulations under investigation lead to very low allowable exposure times (if compared with OEL value), in the order of a few tens of minutes per day. These findings underscore the need to implement effective control measures to protect workers engaged in these activities: it is essential, therefore, to use the most appropriate collective and individual protective equipment, as well as preventive and protective measures when handling and processing material containing RCS.

Several studies, as reported in a recent literature review by Anlimah and collaborators (Anlimah et al. 2023), have highlighted the usefulness of different protective measures such as wet dust suppression methods and on-tool and LEV, for controlling exposures to RCS in the construction trades. The following preventive and protective measures should be taken, among others:

- ▪ Use of local ventilation: the use of local ventilation systems is crucial to capture silica particles (Johnson et al. 2017).
- ▪ Wetting work surfaces: applying water or other wetting agents to work surfaces to reduce the dispersion of silica dust is an effective dust control measure (NIOSH 2002; Bartoli et al. 2012; Healy et al. 2014; Johnson et al. 2017; Weller et al. 2024).
- ▪ Regular cleaning of work areas: regular cleaning of work areas with industrial vacuum cleaners equipped with HEPA filters (Bartoli et al. 2012; Salamon et al. 2021).
- ▪ Worker training: workers must be properly trained and informed about the risks associated with silica exposure and the correct safety procedures to be used; training should include the proper use of PPE and dust control equipment.
- ▪ Appropriate use (i.e. fit testing) of respirators: the selection of respiratory protective equipment, such as FFP2 (N95) and FFP3 (N99) respirators.

It should be emphasized that, although the choice to use PPE is not the optimal one (in conceptual terms, it does not follow a hierarchical system of controls, as stipulated by European Directive 98/24/EC, which provides for (i) elimination of hazardous substances; (ii) substitution by a less hazardous substance; (iii) use of engineering controls at source, including LEV; (iv) administrative controls; and (v) individual protection measures including PPE; in some cases, it is the most practical and applicable solution, if not the only one practicable. The use of PPE is the least likely to be effective in the long term.

Although cutting and grinding tasks are often performed at installation sites, the use of field-portable tools equipped with on-tool wetting systems or LEV can represent an effective and practical solution to reduce exposure to RCS. Promoting the use of such tools on-site is both feasible and advisable, especially when other control measures are not available. Nevertheless, the consistent and optimal implementation of these control systems is generally easier to achieve in fixed and properly equipped environments (e.g. factories), where conditions are more controllable. Therefore, whenever possible, the majority of cutting and grinding operations should be performed in such controlled settings, based on accurate measurements taken at the installation site. Any additional adjustments required on-site should be kept to a minimum and carried out with the use of cutting and grinding tools equipped with wetting systems or on-tool LEV and with effective PPE.

The simulations (Tables S2 and S3) showed that inhaled exposure concentrations can be reduced by using respiratory protection. However, it is important to underline that PPE should be considered the last resort, because PPE can fail or not be used properly, and therefore does not provide as reliable a level of protection. In addition, training of workers on the correct use of PPE and safety practices is crucial to ensure effective protection against RCS exposure. Bystander workers, not just those directly involved processing tiles, could also be exposed to elevated levels of RCS. Although simulation reported in this study shows that high-efficiency respirators can substantially reduce RCS exposure, this measure should not be considered sufficient on its own. As also emphasized in international guidance, reliance on PPE alone is not acceptable for long-term exposure control. Engineering controls such as wet cutting methods and LEV must be prioritized whenever possible, in accordance with the hierarchy of controls.

An important issue to consider is the mode of action of quartz on human lungs. It has been observed for many years that different form of quartz may show different toxicity for the lung (Donaldson and Borm 1998). In particular, it seems that the biological activity of quartz dust is not due to crystallinity but to crystal fragmentation, when conchoidal fractures are formed: beside radical generation, modified surface components of quartz interact with cell membranes inducing both cellular toxicity and inflammatory response (Turci et al. 2016). Free silanols seem particularly important for the interaction between silica and cellular membranes (Pavan et al. 2023). Studies into the toxicity of freshly made dust from quartz and amorphous silica (found in recycled glass) found that both “show irregular particles with sharp edges, stable surface radicals, and sustained release of HO(*) radicals via a Fenton-like mechanism” (Ghiazza et al. 2010) and that amorphous silica behaves such as quartz dust (Pavan et al. 2017). Thus, the presence of glass in the tiles may not be benign.

This study presents several limitations that should be acknowledged and considered in future studies. First, since this study represents a simulation, a small number of measurements were conducted, especially considering the diverse types of material and task performed: while different materials (tile type and thickness) and processes (cutting and grinding) were included in the simulations, the sample size was not sufficient to allow for formal statistical comparisons between these factor. While trends in RCS exposure levels were observed, their relative impact could not be quantified in this study. Future studies with larger datasets should explore the individual and interactive effects of these variables using appropriate statistical models. Moreover, the calculations were conducted without considering the likely combination of tasks performed together.

As mentioned, more in-depth studies on exposure to RCS in unpredictable and intermittent working conditions should be performed, following the standard methodologies of analysis. It would then be useful to investigate, also through other methods, such as via Scanning Electron Microscopy analysis (Carrieri et al. 2020), the characteristics of silica particles, in terms of chemical composition, morphology, and particle size distribution (Contessi et al. 2025). Future studies should also consider the evaluation of chemical composition of the materials being processed (e.g. via Scanning Electron Microscopy analysis), particularly in terms of the potential presence of additional substances.

This study has some strengths. First, the study represented a simulation of real-world working conditions, performed outdoors, without the use of LEV. The data obtained from the simulation (collected following the most up-to-date standard methodologies) were used to estimate the exposure of workers, for different periods of working time with and without PPE.

## 6. Conclusions

The results of this study show that, during the processing of ceramic tile benchtops containing RCS (maximum: 30.5% w/w in bulk samples; 13.5% in respirable dust samples), the exposure of workers during a simulation (2 h of cutting or grinding), can be very high exceeding OELs. The assumption of this work was that (i) the cutting and grinding times are not always necessarily equal to 2 h and that (ii) these processes not characterized by preestablished and continuous processing times.

For these reasons, it is important to carefully evaluate the duration of exposure to RCS during the various tasks and activities performed, as these may vary depending on several factors (e.g. worker experience; tools used; size and type of material processed; etc.). Similarly, it is important to evaluate carefully the nature of the material processed in terms of composition and thickness, as their characteristics could potentially influence workers’ exposure conditions.

Calculations show that the use of PPE may reduce exposure to below relevant OELs, but this is not the preferred protection under the hierarch of controls.

In addition, exposure to RCS may be influenced by the particular type of material processed, in terms of composition and thickness.

## Supplementary material

Supplementary material is available at *Annals of Work Exposures and Health* online.

wxaf044_suppl_Supplementary_Table_S1-S3

## Data Availability

Additional data are available from the corresponding author on reasonable request.
