# Supplementary material for: Evaluation of occupational exposure to airborne quartz in the cutting and grinding of ceramic tiles
Source: Ann Work Expo Health. 2025 Sep 16;70(1):wxaf044. doi: 10.1093/annweh/wxaf044 (PMC12822604; doi:10.1093/annweh/wxaf044)
Supplement: wxaf044_suppl_Supplementary_Table_S1-S3 [file wxaf044_suppl_supplementary_table_s1-s3.docx]

**Evaluation of occupational exposure to airborne quartz in the cutting and grinding of ceramic tiles**

Francesca Borghi^1*+^, Francesca Graziosi^1*+^, Silvia Contessi^1*^; David C. Christiani^2^, Francesco Decataldo^1^, Deborah Glass^3^, Francesco S. Violante^1,4^

^1^Occupational Medicine Unit, Department of Medical and Surgical Sciences, Alma Mater Studiorum University of Bologna, Bologna (Italy)

^2^Harvard Medical School and Harvard TH Chan School of Public Health - Boston (Massashusetts, USA)

^3^Monash School of Public Health and Preventive Medicine - Melbourne (Australia)

^4^Division of Occupational Medicine, IRCCS Azienda Ospedaliero-Universitaria di Bologna, Bologna (Italy)

* These authors contributed equally to this work

^+^ Corresponding authors: FG (francesca.graziosi@unibo.it)

Table S1. Gravimetric analyses performed pre- and post-sampling. Each individual sample was collected while processing a single tile type and thickness.

| **Dust in the Respirable fraction (mg/membrane)** | | | | | | | | | | |
| --- | --- | --- | --- | --- | --- | --- | --- | --- | --- | --- |
|  |  |  | **N*** | **Min.** | **5^th^ per.** | **Mean** | **Median** | **95^th^ per.** | **Max.** | **SD** |
|  |  |  |  |  |  |  |  |  |  |  |
|  |  | *Total by material^+^* | | | | | | | | |
| Personal Samples |  | *W12 mm + W20 mm^+^* | *7* | *0.069* | *0.103* | *0.273* | *0.244* | *0.425* | *0.444* | *0.122* |
|  |  | *B12 mm + B20 mm^+^* | *7* | *0.272* | *0.281* | *0.876* | *0.731* | *1.640* | *1.715* | *0.567* |
|  |  | Detailed Material | | | | | | | | |
|  |  | W12 mm | 4 | 0.183 | 0.188 | 0.254 | 0.230 | 0.354 | 0.373 | 0.072 |
|  |  | W20 mm | 3 | 0.069 | 0.100 | 0.298 | 0.380 | 0.438 | 0.444 | 0.164 |
|  |  | B12 mm | 3 | 0.302 | 0305 | 0.700 | 0.334 | 1.352 | 1.465 | 0.541 |
|  |  | B20 mm | 4 | 0.272 | 0.341 | 1.007 | 1.021 | 1.654 | 1.715 | 0.550 |
|  |  | Activity |  |  |  |  |  |  |  |  |
|  |  | Cutting | 7 | 0.215 | 0.262 | 0.843 | 0.444 | 1.640 | 1.715 | 0.580 |
|  |  | Grinding | 7 | 0.069 | 0.103 | 0.305 | 0.272 | 0.612 | 0.731 | 0.192 |
|  | **Total** | | **14** | **0.069** | **0.143** | **0.574** | **0.354** | **1.553** | **1.715** | **0.509** |
|  |  |  | | | | | | | | |
|  |  | *Total by material^+^* | | | | | | | | |
| Environmental Samples |  | *W12 mm + W20 mm^+^* | *13* | *0.034* | *0.036* | *0.102* | *0.067* | *0.256* | *0.450* | *0.104* |
|  |  | *B12 mm + B20 mm^+^* | *16* | *0.046* | *0.050* | *0.095* | *0.092* | *0.160* | *0.164* | *0.036* |
|  |  | Detailed Material | | | | | | | | |
|  |  | W12 mm | 7 | 0.034 | 0.042 | 0.083 | 0.087 | 0.121 | 0.126 | 0.029 |
|  |  | W20 mm | 6 | 0.038 | 0.040 | 0.124 | 0.058 | 0.362 | 0.450 | 0.147 |
|  |  | B12 mm | 8 | 0.046 | 0.048 | 0.076 | 0.072 | 0.112 | 0.119 | 0.024 |
|  |  | B20 mm | 8 | 0.069 | 0.070 | 0.115 | 0.109 | 0.162 | 0.164 | 0.036 |
|  | **Total** | | **29** | **0.034** | **0.040** | **0.098** | **0.087** | **0.162** | **0.450** | **0.075** |

+The rows in italics indicate the total number of samples collected for each material (Black: B and White: W), combining both 12 mm and 20 mm thicknesses. No combined processing of different thicknesses or materials was performed during a single sampling session.

* Valid N

Table S2. (a) Time-weighted exposure calculation, for cutting and grinding tasks, considering a APFs (Assigned Protection Factors) equal to 10. (b) Time-weighted exposure calculation, for cutting and grinding tasks, considering a APFs (Assigned Protection Factors) equal to 20.

| **(a)**  **APF: 10** |  | **CUTTING** | | | | | | | | | | | |
| --- | --- | --- | --- | --- | --- | --- | --- | --- | --- | --- | --- | --- | --- |
|  |  |  | | | | | | | | | | | |
|  | Minutes/Day | 20 | 30 | 40 | 60 | 90 | 120 | 180 | 240 | 300 | 360 | 420 | 480 |
|  | Mean values [µg/m^3^] | 1.4 | 2.1 | 2.8 | 4.2 | 6.2 | 8.3 | 12.5 | 16,7 | 20.8 | 25.0 | 29.1 | 33.3 |
|  | Median values [µg/m^3^] | 0.7 | 1.0 | 1.4 | 2.1 | 3.1 | 4.1 | 6.2 | 8.2 | 10.3 | 12.3 | 14.4 | 16.4 |
|  | Max. values [µg/m^3^] | 3.7 | 5.6 | 7.4 | 11.1 | 16.7 | 22.2 | 33.3 | 44.5 | 55.6 | 66.7 | 77.8 | 88.9 |
|  |  | **GRINDING** | | | | | | | | | | | |
|  |  |  | | | | | | | | | | | |
|  | Minutes/Day | 20 | 30 | 40 | 60 | 90 | 120 | 180 | 240 | 300 | 360 | 420 | 480 |
|  | Mean values [µg/m^3^] | 0.6 | 0.9 | 1.2 | 1.9 | 2.8 | 3.7 | 5.6 | 7.4 | 9.3 | 11.2 | 13.0 | 14.9 |
|  | Median values [µg/m^3^] | 0.5 | 0.8 | 1.1 | 1.6 | 2.4 | 3.2 | 4.8 | 6.4 | 8.0 | 9.6 | 11.2 | 12.8 |
|  | Max. values [µg/m^3^] | 1.7 | 2.6 | 3.5 | 5.2 | 7.8 | 10.4 | 15.6 | 20.8 | 26.0 | 31.2 | 36.4 | 41.6 |
|  |  |  |  |  |  |  |  |  |  |  |  |  |  |
|  |  |  |  |  |  |  |  |  |  |  |  |  |  |
| **(b)**  **APF: 20** |  | **CUTTING** | | | | | | | | | | | |
|  |  |  | | | | | | | | | | | |
|  | Minutes/Day | 20 | 30 | 40 | 60 | 90 | 120 | 180 | 240 | 300 | 360 | 420 | 480 |
|  | Mean values [µg/m^3^] | 0.7 | 1.0 | 1.4 | 2.1 | 3.1 | 4.2 | 6.2 | 8.3 | 10.4 | 12.5 | 14.6 | 16.7 |
|  | Median values [µg/m^3^] | 0.3 | 0.5 | 0.7 | 1.0 | 1.5 | 2.1 | 3.1 | 4.1 | 5.1 | 6.2 | 7.2 | 8.2 |
|  | Max. values [µg/m^3^] | 1.9 | 2.8 | 3.7 | 5.6 | 8.3 | 11.1 | 16.7 | 22.2 | 27.8 | 33.3 | 38.9 | 44.5 |
|  |  | **GRINDING** | | | | | | | | | | | |
|  |  |  | | | | | | | | | | | |
|  | Minutes/Day | 20 | 30 | 40 | 60 | 90 | 120 | 180 | 240 | 300 | 360 | 420 | 480 |
|  | Mean values [µg/m^3^] | 0.3 | 0.5 | 0.6 | 0.9 | 1.4 | 1.9 | 2.8 | 3.7 | 4.6 | 5.6 | 6.5 | 7.4 |
|  | Median values [µg/m^3^] | 0.3 | 0.4 | 0.5 | 0.8 | 1.2 | 1.6 | 2.4 | 3.2 | 4.0 | 4.8 | 5.6 | 6.4 |
|  | Max. values [µg/m^3^] | 0.9 | 1.3 | 1.7 | 2.6 | 3.9 | 5.2 | 7.8 | 10.4 | 13.0 | 15.6 | 18.2 | 20.8 |

Key. Green: 0-25 µg/m^3^ (< TLV ACGIH Exposure Limit); Yellow: 25-50 µg/m^3^ (< OSHA Permissible Exposure Limit); Orange: 50-100 µg/m^3^ (<: European Exposure Limit).

Table S3. Maximum minutes of tasking per day (cutting/ grinding) not to exceed the limit of occupational exposure considering a APFs (Assigned Protection Factors) equal to 10 (Table S3(a)) and 20 (Table S3(b)).

| **(a) APF=10** | | | | | | | |
| --- | --- | --- | --- | --- | --- | --- | --- |
| Exposure Limit | | CUTTING (min) | | | GRINDING (min) | | |
| Source | µg /m^3^ | Mean | Median | Maximum | Mean | Median | Maximum |
| ACGIH TLV | 25 | 360 | >480 | 135 | >480 | >480 | 288 |
| OSHA PEL | 50 | >480 | >480 | 270 | >480 | >480 | >480 |
| European OEL | 100 | >480 | >480 | >480 | >480 | >480 | >480 |

| **(b) APF=20** | | | | | | | |
| --- | --- | --- | --- | --- | --- | --- | --- |
| Exposure Limit | | CUTTING (min) | | | GRINDING (min) | | |
| Source | µg /m^3^ | Mean | Median | Maximum | Mean | Median | Maximum |
| ACGIH TLV | 25 | >480 | >480 | 270 | >480 | >480 | >480 |
| OSHA PEL | 50 | >480 | >480 | >480 | >480 | >480 | >480 |
| European OEL | 100 | >480 | >480 | >480 | >480 | >480 | >480 |
